# Unlocking the Biochemical Potential of *Diadema setosum* Tests: A Pathway Toward Circular Marine Bioeconomy

**DOI:** 10.3390/molecules30183700

**Published:** 2025-09-11

**Authors:** Bilge Bilgin Fıçıcılar, Koray Korkmaz

**Affiliations:** Fatsa Faculty of Marine Sciences, Department of Fisheries Technologies, Ordu University, Fatsa, 52400 Ordu, Turkey; koraykorkmaz@odu.edu.tr

**Keywords:** *Diadema setosum*, sea urchin, chemical composition, blue economy

## Abstract

This study investigates the biochemical and elemental composition of the test of *Diadema setosum* (*D. setosum*)*,* a sea urchin species increasingly processed in Turkey, where the shell is commonly treated as industrial waste. Specimens were collected from the Mediterranean and Aegean Seas, and the test material was subjected to amino acid profiling, protein quantification, and X-ray fluorescence (XRF) analysis. The results revealed a considerable protein content (8.03%) and a rich amino acid spectrum dominated by glycine, aspartic acid, and arginine, supporting the presence of residual structural proteins even after processing. Mineral analysis showed a high calcium oxide concentration (43.19%), alongside significant levels of magnesium, phosphorus, strontium, and trace elements such as zinc, copper, and molybdenum. Rare earth elements and radionuclides including neodymium, samarium, and uranium were also detected, suggesting sediment interaction. These findings suggest that *D. setosum* tests could represent a sustainable source of bioavailable minerals and proteinaceous material, with prospective applications in fish or livestock feed, hydroxyapatite synthesis, or calcium oxide production, pending further validation.

## 1. Introduction

*D. setosum* is a long-spined sea urchin belonging to the phylum *Echinodermata* and the class *Echinoidea*, easily recognized by its dark-colored body, long venomous spines, and a distinct orange ring around the anal cone [1].

*D. setosum* exhibits a broad geographic distribution throughout the Indo-West Pacific region, encompassing the Red Sea (including the Gulf of Suez and the Gulf of Aqaba), the eastern coast of Africa, and extending eastward to Japan and Australia [2,3]. This cryptic echinoid species is typically associated with coral reef ecosystems and shallow rocky substrates, generally occurring at depths ranging from 1 to 6 m. *D. setosum* was first recorded in Turkish waters in 2006, near Kaş along the southwestern coast [4]. Since then, it has rapidly expanded its distribution, with subsequent reports along the Mediterranean coast in Hatay [5], the Aegean Sea [6,7], and even the Marmara Sea [8]. This spread is believed to be the result of Lessepsian migration, with the species entering the Mediterranean through the Suez Canal [4].

**Figure 1:**
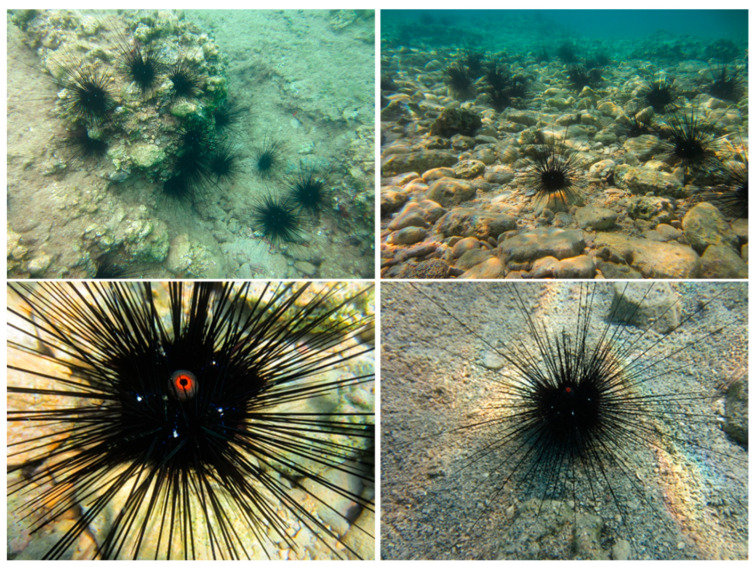
Underwater view of *Diadema setosum* (photograph taken by the authors).

*D. setosum* is commonly found in shallow sublittoral zones, at depths ranging from 1 to 20 m, with the highest concentrations typically recorded between 4 and 6 m. The species primarily inhabits rocky areas, where individuals seek shelter in crevices and beneath overhangs, particularly during periods of intense sunlight. It has also been observed in other environments, such as sandy bottoms and seagrass meadows. Morphologically, *D. setosum* is characterized by its elongated, venomous spines and a rigid, dark-colored test, which generally measures 6–7 cm in diameter and 3.5–4 cm in height (Figure 1). The spines serve important functions in both defense and movement and are among the most visually distinctive features of the species. The average lifespan is approximately 3.5 years, with mature individuals weighing between 35 and 80 g [9].

Although *D. setosum* is primarily studied for its ecological impact, its roe is consumed in various parts of the world, particularly in countries such as Japan, Italy, and France, as well as in regions of North and South America, including New York, Boston, California, and British Columbia, where it is considered a delicacy. The edible portion comprises the gonadal lobes, roe in females and milt in males, both of which are prized in raw and fermented culinary forms. In Turkey, the roe is manually extracted from the shell and sold as a premium seafood product. However, only about 12% of the total body mass is composed of edible roe, while the remaining 88% consists of the test and protective spines, which are discarded during processing. Export data from 2020 to 2024 reflects a growing demand for Turkish sea urchin roe, with exports peaking in 2022 at over 130,000 kg and generating more than €8.3 million primarily to markets in the European Union. From over 130,000 kg of *D. setosum* harvested annually, approximately 80% is commonly discarded as waste. This shows that the species is gaining growing importance for industry and the market, especially in the Mediterranean, where it holds both economic value and regional significance.

*D. setosum* is commercially harvested in Turkish waters primarily for its roe, which is exported to markets in Italy, Greece, and Spain, generating significant economic value [10]. While previous studies have addressed the nutritional composition of the gonads [11,12] and investigated potential uses of sea urchin waste in other species, a targeted review of scientific databases revealed no published research on the valorization of *D. setosum* test as a waste material. This lack of data represents a clear knowledge gap, particularly in the context of circular economy strategies for marine bioresources and the sustainable management of invasive species in the Mediterranean basin.

In this study, we aimed to investigate the potential for valorizing this discarded biomass by conducting amino acid profiling (both free and total) using Liquid Chromatography—Tandem Mass Spectrometry (LC-MS/MS), Carbon, Hydrogen, Nitrogen, Sulfur (CHNS) elemental analysis to determine its organic composition and X-ray fluorescence (XRF) to assess its inorganic elemental content. The findings of this study are expected to facilitate the development of novel industrial applications for *D. setosum* by-products, extending its relevance beyond food use and aligning with sustainable blue economy initiatives in the region.

## 2. Results and Discussion

### 2.1. Proximate Composition

In *D. setosum*, the edible portion constitutes approximately 20% of the total body weight, while the remaining 80%, primarily composed of the test and spines, is generally discarded as waste. In comparison, the tests with movable spines of red (*Strongylocentrotus franciscanus*) and green (*Strongylocentrotus droebachiensis*) sea urchins account for around 47.9 ± 5.6% and 40.7 ± 3.3% of their total body weights, respectively [13].

The moisture content of the *D. setosum* test was measured as 2.56 ± 0.10%, which is very similar to the value reported for *Strongylocentrotus franciscanus* (2.52 ± 0.24%) and slightly higher than *S. droebachiensis* (1.74 ± 0.03%) [13]. These low moisture values are consistent with the mineralized structure of sea urchin tests [13,14]. In this study, the ash content of the *D. setosum* test was 84.85 ± 0.06%, confirming that mineral matter constitutes the predominant component of the test (Table 1) [13]. Drozdov, et al. [15] reported that the mineral content (expressed as ash) in sea urchin tests and spines ranged from 51.69% to 88.95%, with generally higher values observed in the tests compared to the spines. The test of *D. setosum* contained a significantly higher amount of protein (8.03%) compared to *Strongylocentrotus franciscanus* and *S. droebachiensis*, which had 4.06% and 4.99%, respectively (Table 1). The lipid content of *D. setosum* was determined as 0.72 ± 0.07%, indicating the presence of lipids in trace amounts within the test. Similar values have been reported in other sea urchin species; Drozdov, Sharmankina, Zemnukhova and Polyakova [15] determined lipid levels between 0.65% and 0.76% in the tests of *Strongylocentrotus intermedius*. Swift, et al. [16] and Kanold, et al. [17] also reported lipid contents below 1% of the total dry weight in the organic matrix of sea urchin tests. The nitrogen content of the *D. setosum* test was 1.32 ± 0.02%, indicating the presence of organic material, primarily associated with proteins, in the test structure. This value is higher than those reported in other sea urchin species. Amarowicz, Synowiecki and Shahidi [13] reported nitrogen solubility values of 0.65 ± 0.02 g/100 g for *Strongylocentrotus franciscanus* and 0.79 ± 0.05 g/100 g for *S. droebachiensis* test samples. The comparatively higher nitrogen level in *D. setosum* may be attributed to species-specific characteristics or the retention of protein-rich organic matrix within the test (Table 1).

To date, no data has been reported on the carbohydrate content of the sea urchin test. A study on red (*Strongylocentrotus franciscanus*) and green (*S. droebachiensis*) sea urchins found no detectable chitin and only trace levels of glucosamine in the test matrix, indicating that the carbohydrate fraction is typically negligible [13]. In the present study, the carbohydrate content of *D. setosum* tests was comparatively higher. This result is likely related to the collection method, as tests obtained after roe separation in processing facilities may retain small amounts of gonadal tissue and other soft parts, which can contribute to elevated carbohydrate values despite cleaning.

### 2.2. Amino Acid Composition

The total and free amino acid profiles of *Diadema setosum* test hydrolysate are presented in Table 2.

The amino acid composition of the *D. setosum* test was compared with previously reported data for *Strongylocentrotus intermedius* roe and *Strongylocentrotus franciscanus* test proteins [13]. In terms of total amino acids (expressed as g/100 g protein), the *D. setosum* test was particularly rich in glycine (11.83), aspartic acid (9.37), and glutamic acid (9.25), which are commonly associated with the organic matrix of calcified structures. These levels were comparable to those found in the *S. franciscanus* test (glycine: 12.21; aspartic acid: 8.93; glutamic acid: 12.95), indicating similar structural protein patterns. In contrast, the roe proteins of *S. intermedius*, which serve a nutritional role, exhibited a broader balance of essential amino acids with relatively lower glycine (4.2 g/100 g) and higher levels of lysine and histidine [18]. Notably, *D. setosum* also showed elevated concentrations of arginine (8.29) and alanine (5.57), which are typical of collagen-like and mineral-associated proteins. These results indicate that while the *D. setosum* test shares compositional similarities with other echinoid tests, it also presents a distinct amino acid profile reflective of its structural and potentially biofunctional properties.

### 2.3. Elemental Composition

**Figure 2:**
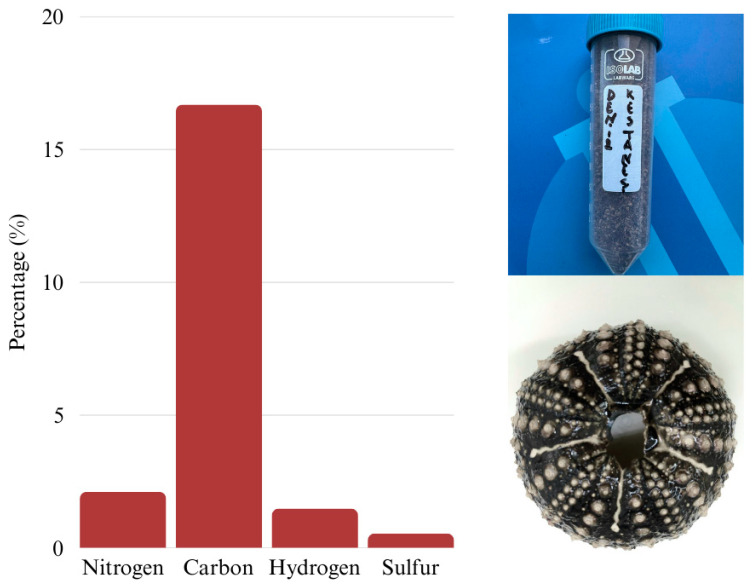
Elemental composition of *Diadema setosum* test.

The elemental composition analysis of the *D. setosum* test revealed measurable levels of organic elements, supporting the presence of residual organic matter within the test matrix. On average, the sample contained 2.12% nitrogen, 16.70% carbon, 1.49% hydrogen, and 0.55% sulfur. The relatively high nitrogen and carbon contents are consistent with the presence of proteinaceous material, which aligns with the previously measured 8.03% protein content in the same test sample (Figure 2). These findings indicate that, despite the predominantly mineral structure of sea urchin tests, a notable amount of protein remains associated with the test, likely as part of the organic matrix embedded within the calcified layers.

The elemental composition of the *D. setosum* test, determined by X-ray fluorescence (XRF) analysis, reveals that calcium oxide (CaO) is the dominant component, accounting for 43.19% of the total amount (Table 3). This is accompanied by a notably high ignition loss (51.07%), which likely reflects the decomposition of calcium carbonate (CaCO_3_) into carbon dioxide during thermal analysis, a common characteristic of marine echinoderm tests primarily composed of high-magnesium calcite or aragonite [19,20,21]. Minor constituents include magnesium oxide (MgO, 2.17%) and sodium oxide (Na_2_O, 1.57%), both typical of marine biominerals and possibly indicative of dolomitic substitution or ionic incorporation during test formation. The detection of chlorine (Cl, 1.43%) suggests residual sea salt or surface-bound marine compounds, despite cleaning procedures. Trace amounts of silicon dioxide (SiO_2_, 0.30%), aluminum oxide (Al_2_O_3_, 0.06%), and iron oxide (Fe_2_O_3_, 0.11%) may originate from environmental exposure or minimal sediment contamination. Additionally, the presence of phosphorus pentoxide (P_2_O_5_, 0.24%) could signal remnants of organic material or phosphate inclusions within the test matrix.

When compared to other echinoid species, the *D. setosum* test exhibits comparable CaO content to *S. intermedius* (45.98%) but shows substantially lower MgO (11.03%) and Na_2_O (40.70%) levels. In contrast, *Mesocentrotus nudus* presents even higher concentrations of CaO (68.14%) and MgO (19.49%), highlighting interspecies or environmental differences in test mineralization. Overall, the chemical profile aligns with previously reported echinoid data, reaffirming the predominance of carbonate minerals, with minor contributions from both inorganic marine residues and organic components [22].

The trace element analysis of the *D. setosum* test reveals a complex mineral profile with significant biological and environmental implications. Strontium (Sr) was found in exceptionally high concentrations (2729.00 ppm), which is consistent with its known behavior as a calcium analog frequently incorporated into echinoderm skeletons during biomineralization (Table 4) [23]. Elevated Sr levels are typical in marine carbonate structures and support the characterization of the test as a high-magnesium calcitic material. Strontium readily substitutes for calcium in the carbonate lattice due to its similar ionic radius, especially in organisms that produce high-magnesium calcite, such as sea urchins. This substitution is a well-documented feature of echinoderm biomineralization and contributes to the mechanical properties and crystal morphology of their endoskeletons [24]. Elevated Sr levels are also influenced by ambient seawater composition and the organism’s physiological regulation during skeletal formation. The values reported here align closely with previous studies, which found that echinoderms can accumulate Sr in concentrationsranging from several hundred to several thousand ppm in their tests and spines.

The presence of zinc (Zn, 38.3 ppm) and copper (Cu, 9.7 ppm) at moderate levels likely reflects their biological roles as essential trace elements, particularly as cofactors in enzymatic systems and immune function. Nickel (Ni, 30.0 ppm) and molybdenum (Mo, 2.7 ppm), although not core structural elements, may also be involved in metabolic pathways or incorporated passively during skeletal formation. Nickel concentrations in the test of *D. setosum* were reported at 2.390 mg/kg in İskenderun and 2.914 mg/kg in Arsuz, indicating moderate accumulation levels consistent across both sites (Table 4) [25].

Lead (Pb, 63.5 ppm) and arsenic (As, 5.6 ppm) are of environmental concern, as they have no known biological function and are typically associated with anthropogenic pollution. Their accumulation in the test may indicate exposure to contaminated sediments or industrial runoff, underscoring the importance of biomonitoring in coastal regions. Lead was reported at relatively low concentrations in the sea urchin tests of *Sphaerechinus granularis* and *Paracentrotus lividus*, with values of around 0.89 and 0.94 mg/kg, respectively [26]. The elevated Pb levels detected in the test of *Diadema setosum* in the present study are in agreement with earlier reports on this species. Al-Najjar, et al. [27] reported concentrations exceeding 40 ppm Pb in the spines and tests of *D. setosum* from the Gulf of Aqaba, supporting the view that this species is prone to heavy metal accumulation in its calcified structures. The mechanism of Pb enrichment is thought to involve adsorption onto the carbonate skeleton or partial substitution for Ca^2+^ and Mg^2+^ within the mineral lattice [28,29]. Similar patterns have been observed in other echinoderms, such as *Asterias rubens*, where skeletal tissues were proposed as sensitive indicators of Pb and Cd contamination [30]. In the present study, the Pb concentration (63.5 ppm) was higher than the values reported by Al-Najjar, Al Tawaha, Wahsha and Hilal [27], which may reflect differences in local contamination sources or environmental conditions. Although As was present at lower concentrations than Pb, its detection remains ecologically relevant, given its recognized toxicity and ability to enter marine food webs [31,32]. Understanding these contaminants is important in order to better assess their environmental and ecological risks. When assessed in relation to existing regulatory standards, the concentrations of Pb and As detected in *D. setosum* are of particular concern. EC [33] specifies maximum permissible levels of 0.50 mg/kg Pb in crustaceans and 1.50 mg/kg Pb in bivalve mollusks, while the TFC [34] establishes comparable thresholds. In contrast, no maximum levels for total arsenic or inorganic arsenic have yet been set in fish and fishery products under the EU legislation. Nevertheless, the 5.6 mg/kg total As measured in *D. setosum* should be viewed in light of the recent EFSA [35], which set a benchmark dose lower confidence limit (BMDL) of 0.06 µg/kg bw/day for inorganic As, indicating that even low-level chronic exposures may pose health risks. The values reported here for *D. setosum* (63.5 mg/kg Pb; 5.6 mg/kg As) therefore highlight a substantial safety issue. Although the calcified test is not a direct food source, any prospective valorization for nutraceutical, cosmetic, or industrial purposes would necessitate rigorous toxicological evaluation and the implementation of effective decontamination strategies.

Rare earth elements such as neodymium (135.5 ppm) and samarium (5.8 ppm), together with zirconium (124.4 ppm) and barium (90.8 ppm), were found in relatively high concentrations in the test of *D. setosum*. These elements are not typically incorporated through active biological processes but are more often associated with detrital input, sediment interaction, or adsorption onto mineral surfaces. Their presence suggests that *D. setosum* may be in frequent contact with particulate-rich sediments, possibly due to its benthic feeding behavior or habitat preference near the seabed.

The detection of uranium (9.8 ppm) and thorium (29.8 ppm), although present at lower levels, is still notable. These naturally occurring radionuclides are usually found in trace amounts in seawater and marine sediments. Their accumulation in the test may point to long-term exposure in the surrounding environment or passive incorporation during test formation, reflecting both the mineral makeup of the habitat and potential anthropogenic inputs.

## 3. Materials and Methods

### 3.1. Materials

Specimens of *D. setosum* were collected by divers primarily from the Mediterranean Sea and the Aegean Sea. A total of 1000 randomly selected *D. setosum* were caught. Following collection, the individuals were transported to a processing facility where the roe was carefully extracted. The collected *D. setosum* tests contained approximately 4–5% spines by weight, which were manually removed and discarded. The remaining tests were cleaned under running tap water, sun-dried for three days at ambient temperature, mechanically ground, and sieved through a 150 µm mesh to obtain a uniform fine powder for further analysis. The resulting powders were vacuum-packed in polyethylene pouches and stored frozen at −20 °C until use.

### 3.2. Proximate Analysis

Moisture content was measured by drying the samples in an oven (Termal, Istanbul, Türkiye) at 105 °C for 8 h, based on the method described by AOAC [36]. Total nitrogen and ash contents were determined according to references [37,38]. Total lipids were extracted using a chloroform–methanol–water mixture, following the method of Bligh and Dyer [39]. Total carbohydrate content was determined using the phenol–sulfuric acid method [40].

### 3.3. Amino Acid Analysis

The amino acid content in the *D. setosum* test was determined as both total and free amino acids. A modified version of the methods was developed by Lee and Hwang [41]. Chan and Matanjun [42] used an LC-MS/MS system (Thermo Fisher Scientific Inc., Waltham, MA, USA). For analysis, 0.2 g of homogenized sample was mixed with 10 mL of 6 M hydrochloric acid (Sigma Aldrich, St. Louis, MO, USA). The solution was sealed, vortexed for 5 min, and then hydrolyzed by heating in an oven at 110 °C for 24 h. After hydrolysis, the mixture was cooled to room temperature and centrifuged (Model 5810 R, Eppendorf AG, Hamburg, Germany) at 4000 rpm for 15 min at 4 °C. The resulting supernatant was filtered through a 0.45 μm PTFE membrane (MilliporeSigma, Burlington, MA, USA) before analysis. Free amino acids were quantified from untreated homogenized samples following the same extraction, centrifugation, and filtration steps without hydrolysis.

### 3.4. Elemental Characterization

Elemental analysis of ground *D. setosum* test samples was carried out using two complementary analytical techniques: wavelength dispersive X-ray fluorescence (WD-XRF) and CHNS elemental analysis.

For inorganic elemental profiling, WD-XRF analysis was performed using a PANalytical Axios Max spectrometer (Malvern PANalytical, Almelo, The Netherlands). Pre-ground test samples were pressed into pellets with boric acid (Sigma-Aldrich, St. Louis, MO, USA) as a binder. Elemental concentrations were determined using the fundamental parameters (FP) method under vacuum conditions. Certified reference materials were used to ensure accuracy in calcium carbonate-rich matrices.

To assess the organic elemental composition, C, N, S, and H contents were measured using a Thermo Scientific Flash 2000 elemental analyzer (Thermo Fisher Scientific, Waltham, MA, USA). Approximately 2 mg of each pre-ground sample was weighed and combusted in an oxygen-rich environment at 950–1000 °C. The resulting combustion gases were separated and quantified, allowing precise determination of carbon (C), nitrogen (N), sulfur (S), and hydrogen (H) percentages. Following elemental analysis, Loss on Ignition (LOI) was performed to estimate volatile components such as moisture, organics, and carbonate content. Approximately 1 g of each ground sample was placed in a porcelain crucible (Isolab Laborgeräte GmbH, Eschau, Germany) and heated in a muffle furnace (Protherm Furnaces, Ankara, Turkey) at 900 °C for 6 h. The weight loss was recorded and expressed as a percentage of the initial mass [41,43].

### 3.5. Statistical Analysis

All measurements were performed in triplicate, and results are expressed as mean ± standard deviation (SD). Descriptive statistics (mean and SD) were calculated using SPSS software (IBM SPSS Statistics, version 20.0, IBM Corp., Armonk, NY, USA).

## 4. Conclusions

The present study provides valuable baseline information on the composition of the test of *Diadema setosum*, a species increasingly processed in Turkey for its edible roe and exported as a commercially valuable product that contributes to the national economy. Although typically discarded, the test was shown to contain a substantial amount of protein (8.03%) with an amino acid profile enriched in glycine, aspartic acid, and arginine, as well as notable levels of calcium oxide (43.19%) together with magnesium, sodium, phosphorus, and trace elements such as strontium and zinc. These compositional features suggest that *D. setosum* tests could potentially be explored as a source of proteinaceous and mineral inputs.

The findings indicate that this material merits further investigation for both nutritional applications in aquaculture and animal husbandry and industrial uses such as hydroxyapatite synthesis and bioceramic production, or as a natural CaO precursor in agricultural and chemical processes. By considering such underutilized by-products, this work contributes to ongoing efforts toward sustainable resource management, waste valorization, and the advancement of a circular bioeconomy in marine bioprocessing.

## Figures and Tables

**Table 1:** Proximate composition of *Diadema setosum* test.

Component	Content (%)
Moisture (%)	2.56
Protein (%)	8.03
Lipid (%)	0.72
Ash (%)	84.85
Nitrogen (%)	1.32
Carbohydrate (%)	3.84

Values are expressed as mean (*n* = 3).

**Table 2:** The total and free amino acid composition of *Diadema setosum* test.

Amino Acid	Total Amino Acids (mg/g)	Free Amino Acids (mg/100 g)
Aspartic acid	93.71 ± 0.04	0.71 ± 0.06
Glutamic acid	92.45 ± 0.10	5.12 ± 0.05
Histidine	15.10 ± 0.08	0.60 ± 0.04
Arginine	82.92 ± 0.06	4.65 ± 0.07
Glycine	118.30 ± 0.02	16.43 ± 0.02
Alanine	55.74 ± 0.02	5.31 ± 0.04
Proline	27.40 ± 0.02	0.70 ± 0.04
Methionine	10.62 ± 0.09	0.93 ± 0.05
Tryptophan	1.11 ± 0.06	0.90 ± 0.08
Phenylalanine	31.87 ± 0.07	1.81 ± 0.03
Lysine	78.30 ± 0.01	4.62 ± 0.06
Leucine	58.23 ± 0.10	4.45 ± 0.06
Isoleucine	32.50 ± 0.08	2.92 ± 0.01
Serine	32.68 ± 0.03	1.91 ± 0.06
Valine	45.11 ± 0.03	5.20 ± 0.03
Threonine	38.20 ± 0.03	3.27 ± 0.02
Tyrosine	27.82 ± 0.04	2.82 ± 0.10

Values are presented as mean ± standard deviation (SD), based on triplicate measurements (*n* = 3).

**Table 3:** XRF results of *Diadema setosum* test.

Component	SiO_2_	Al_2_O_3_	Fe_2_O_3_	CaO	MgO	K_2_O	Na_2_O	TiO_2_	MnO	Cl	P_2_O_5_	Ig Loss
Content (%)	0.30	0.06	0.11	43.19	2.17	0.31	1.57	0.02	0.02	1.43	0.24	51.07

Values are expressed as the mean of three replicates (*n* = 3).

**Table 4:** Mineral composition of *Diadema setosum* test.

**Element**	**Sc**	**Ni**	**Cu**	**Zn**	**Ga**	**As**	**Rb**	**Sr**	**Zr**	**Mo**
Content (mg/kg)	22.60	30.00	9.70	38.30	4.60	5.60	19.80	2729.00	124.40	2.70
**Element**	**Cd**	**Ba**	**Nd**	**Sm**	**Yb**	**Hf**	**Pb**	**Th**	**U**	**Sn**
Content (mg/kg)	0.20	90.80	135.50	5.80	0.20	4.90	63.50	29.80	9.80	N.D.

Values are expressed as the mean of three replicates (*n* = 3). N.D. = Not detected.

## Data Availability

The data presented in this study are available on request from the corresponding author.
